# Fellow-Workers and the Roll of Honour

**Published:** 1915-06-05

**Authors:** 


### ROUND THE WORLD.

[The Editor invites Early Information and Contributions Marked "Fellow-Workers."]

### Fellow-Workers and the Roll of Honour.

Dr. Austin Threlfall Nankivell, who leaves Poole temporarily with an R.A.M.C. commission, is one of the most brilliant younger members of the public health service. At "Bart.'s" and King's College he showed great promise in clinical, pathological, and bacteriological work, and his friends were not very surprised when he won the gold medal in State medicine at the M.D. Lond. five years ago. After holding the posts of demonstrator of public health at King's College, resident medical officer to the Isolation Hospital, and bacteriologist to the county borough of Croydon, he went to St. Austell, in Cornwall, as medical officer of health, staying there until appointed to the posts of medical officer of health and school medical officer at Poole.

Dr. William Stewart Stalker, M.D. Glasg., D.P.H. Oxon., graduated M.B., Ch.B. Glasg. in 1899 and served as a junior in the public health departments of East Ham and Wiltshire before being elected to the posts he is now relinquishing temporarily for war service—namely, medical officer of health and school medical officer for Swinton and Pendlebury. Some four years ago Dr. Stalker went into the question of picture palaces being possible centres for the spread of infectious diseases.

Amongst the casualties reported from Egypt one notices with regret the name of Major Albert Hilton, R.A.M.C. (T.), who in civil life was well known at Hurst, near Ashton-under-Lyne, where he had practised for many years. Qualifying L.S.A. from Owens College, Manchester, nineteen years ago, he soon settled down in private practice, and ultimately became medical officer of health for Hurst and surgeon to the Lancashire County Constabulary. Major Hilton's keen interest in the welfare of his profession was rewarded by his election to the presidential chair of the local division of the British Medical Association a few years ago. The better to fit himself for public health work, the late officer took the D.P.H. Cantab.

Whilst assisting a wounded companion on May 22, Lieutenant George Martin Chapman was killed by a shell. His loss is keenly felt by those who knew him in the full force of his kindly and vigorous personality at the London Hospital, where he was house surgeon only a few months ago. Born in New Zealand, he received part of his education at Otago University, whence he proceeded to Cambridge and the "London," qualifying in 1912. Lieutenant Chapman was a sportsman to his finger-tips, and defeated all comers as heavy-weight boxer at his hospital; in addition to which he won his blue at Cambridge for Rugby football, playing subsequently for the London Hospital and for the Harlequins. At Cambridge he gained a half-blue for his boxing prowess. Early this year he was decorated by the French Government in recognition of his bravery in saving life during a wreck off Boulogne.

Military rank will be held at the Springfield War Hospital, whilst serving in connection therewith, by Dr. Reginald Worth and Dr. Gayton Warwick Smith, who are known respectively as medical superintendent and senior assistant medical officer to the Middlesex County Mental Hospital, near Tooting.

Educated at St. Mary's Hospital, Dr. R. Worth, who will be a major, qualified in 1896 and took the M.B., B.S. Durh. five years later. Before being appointed to the charge of the Tooting institution he was assistant house surgeon at Cardiff Infirmary, and clinical assistant at the famous West Riding Asylum, at Wakefield.

Whilst serving at the Napsbury War Hospital, Dr. Lancelot William Rolleston will hold the rank of major in the R.A.M.C. A former student of St. Bartholomew's Hospital, Dr. Rolleston is well known in the Asylum Service as medical superintendent of the big Middlesex county institution for the insane at Napsbury. Qualifying M.B., B.S. Durh. in 1892, he was for a time resident at the Metropolitan Hospital, after which he learnt the routine of asylum work as assistant medical officer at Tooting.

Mr. Arthur O'Neill, L.R.C.P., M.R.C.S., who has been one of Dr. Rolleston's assistant medical officers, will rank as captain whilst working at the Napsbury War Hospital. Also a "Bart.'s" man, he qualified in 1907, and has held the posts of house-surgeon at the Windsor and Eton Royal Infirmary, and assistant R.M.O. to the old North-West London Hospital.

Dr. G. W. Smith, M.D., B.S. Lond., who is to hold a captaincy, qualified from Guy's in 1902, and became a graduate in medicine and surgery of Durham as well as of London University. He also took the D.P.H. Cantab., and was for a time a clinical assistant at Guy's.

Captain Harry Young Cameron Taylor, who holds a commission in the R.A.M.C., is in private life a Bexhill practitioner. Qualifying at Edinburgh University in 1896, he proceeded to hold various resident posts, including those of house surgeon to the Hospital for Sick Children in Great Ormond Street, and to the Huddersfield Infirmary. At the time of the South African trouble he served as civil surgeon with the Field Force. Captain Taylor became an F.R.C.S. Edin. ten years ago.

The committee of the Ulster Eye, Ear, and Throat Hospital, Belfast, have released Dr. W. A. Anderson and Dr. I. A. Davidson for military service; leave of absence having been granted in the first instance for one year, and the times for attendance in the out-patient department at that institution having been curtailed in consequence.

Dr. William Arthur Davidson qualified in London five years ago, and, being a student of Cambridge University, took the M.B., B.C. there last year. He was for a time senior house surgeon to our premier Metropolitan eye hospital (Royal London Ophthalmic), and, on returning to Belfast, where his early studies were pursued, held the posts of house physician and house surgeon at the Royal Victoria Hospital.

Dr. Isaac Alexander Davidson graduated in medicine and surgery in Ireland over twenty years ago, and subsequently became an M.D., in addition to which he took the Cambridge D.P.H. His past appointments include those of house physician and house surgeon to the Royal Victoria Hospital, Belfast, and honorary anæsthetist to that institution, as well as to the Belfast Hospital for Sick Children.

